# Oncogenic miR-19a and miR-19b co-regulate tumor suppressor MTUS1 to promote cell proliferation and migration in lung cancer

**DOI:** 10.1007/s13238-017-0393-7

**Published:** 2017-03-31

**Authors:** Yuanyuan Gu, Shuoxin Liu, Xiaodan Zhang, Guimin Chen, Hongwei Liang, Mengchao Yu, Zhicong Liao, Yong Zhou, Chen-Yu Zhang, Tao Wang, Chen Wang, Junfeng Zhang, Xi Chen

**Affiliations:** 1 State Key Laboratory of Pharmaceutical Biotechnology, Jiangsu Engineering Research Center for Micro, RNA Biology and Biotechnology, NJU Advanced Institute for Life Sciences (NAILS), School of Life Sciences, Nanjing University, Nanjing, 210046 China; 2 The Second Department of Medical Oncology, Linyi Tumor Hospital, Linyi, 276000 China; 3 Department of Cardio-Thoracic Surgery, Nanjing Drum Tower Hospital Affiliated to Medical School of Nanjing University and Nanjing Multi-Center Biobank, Nanjing, 210008 China

**Keywords:** microRNA, MTUS1, miR-19a/b, lung cancer, proliferation, migration

## Abstract

MTUS1 (microtubule-associated tumor suppressor 1) has been identified that can function as a tumor suppressor gene in many malignant tumors. However, the function and mechanisms underlying the regulation of MTUS1 are unclear. In the present study, we reported that miR-19a and miR-19b (miR-19a/b) promote proliferation and migration of lung cancer cells by targeting MTUS1. First, MTUS1 was proved to function as a tumor suppressor in lung cancer and was linked to cell proliferation and migration promotion. Second, an inverse correlation between miR-19a/b expression and MTUS1 mRNA/protein expression was noted in human lung cancer tissues. Third, MTUS1 was appraised as a direct target of miR-19a/b by bioinformatics analysis. Fourth, direct MTUS1 regulation by miR-19a/b in lung cancer cells was experimentally affirmed by cell transfection assay and luciferase reporter assay. Finally, miR-19a/b were shown to cooperatively repress MTUS1 expression and synergistically regulate MTUS1 expression to promote lung cancer cell proliferation and migration. In conclusion, our findings have provided the first clues regarding the roles of miR-19a/b, which appear to function as oncomirs in lung cancer by downregulating MTUS1.

**Electronic supplementary material:**

The online version of this article (doi:10.1007/s13238-017-0393-7) contains supplementary material, which is available to authorized users.

## INTRODUCTION

All over the world, lung cancer has been the leading cause of cancer-related death (Ramalingam et al., 1998). The 56% of lung cancers are diagnosed during the late stages of disease while the early stages of disease are usually asymptomatic; only 15% of cases can be diagnosed during a local stage (Siegel et al., 2012). Many carcinogenic factors, such as smoking, genetic mutations and declining immune function, may increase the risk of developing lung cancer. Understanding the molecular mechanism of lung cancer is the hinge of identifying new therapeutic targets and designing new drugs. However, the exact mechanisms underlying the development of lung cancer remain complex and poorly understood. These obstacles underscore the need for in-depth exploration for genes that are aberrantly expressed during lung carcinogenesis, and the need for intensive investigations of the roles of these genes in tumor biology.

 MTUS1 (microtubule-associated tumor suppressor 1 gene), also known as mitochondrial tumor suppressor gene 1, is localized to chromosome 8p22 and comprises 17 exons (Molina et al., 2011). MTUS1 encodes a family of angiotensin II (AT2) receptor-interacting proteins (ATIP), and by alternative exon utilization in this gene, 5 known transcript variants are coded to 5 different protein isoforms of ATIP (ATIP1, ATIP2, ATIP3a, ATIP3b, and ATIP4) (Di Benedetto et al., 2006). The 5 ATIP protein exhibit distinct motifs in their amino-terminal portion that determine whether they localize to the cell membrane, the cytosol or the nucleus (ATIP1, ATIP3 and ATIP4 localize to the cytosol, nucleus and plasma membrane, respectively). MTUS1 downregulation or loss has been documented in many types of cancer, including colon, oral, gastric, and bladder cancer (Zuern et al., 2010; Ding et al., 2012; Xiao et al., 2012; Li et al., 2014a). Although several papers regarding the relationship between MTUS1 gene expression and cancer have been published, the roles of MTUS1 in the development of human cancers (especially lung cancer) remain unclear. The target of this study was to evaluate the association between MTUS1 gene expression and lung cancer and to identify the molecular pathways associated with MTUS1 regulation.

Over the past years, a class of small, single-stranded, non-coding RNAs, known as microRNAs (miRNAs), have emerged as major regulators of the development of human cancers, including lung cancer (Guz et al., 2014; Kang and Lee, 2014). Downregulation of tumor-suppressor miRNAs (targeting oncogenes) and upregulation of oncogenic miRNAs (targeting tumor suppressor genes) lead to cancer cell dysfunction, including malignant proliferation, invasion, and metastasis (Calin and Croce, 2006; Ma and Weinberg, 2008; Nicoloso et al., 2009). The miR-17-92 miRNA cluster is one of the best characterized groups of miRNA oncogenes, and genomic amplification or aberrant expression of these miRNAs is frequently observed in a variety of tumor types (Olive et al., 2013; Guinot et al., 2016; Robaina et al., 2016). As members of the miR-17-92 cluster, miR-19a and miR-19b (miR-19a/b) usually function as oncogenes in many types of cancer, including gastric cancer (Lu et al., 2015), pancreatic cancer (Wang et al., 2016), and breast cancer (Li et al., 2014b). However, the molecular basis underlying the contributions of miR-19a/b to the development of lung cancer remains to be elucidated.

In this study, we found that MTUS1 functions as a tumor suppressor in lung cancer cells. Next, we identified miR-19a/b as a potential regulator of MTUS1 using bioinformatics analysis and experimentally confirmed that MTUS1 is directly regulated by miR-19a/b in lung cancer cells. Finally, we showed that MTUS1 is synergistically suppressed by miR19a/b, resulting in lung cancer cell proliferation and migration.

## RESULTS

### MTUS1 functions as a tumor suppressor in lung cancer cells

**Figure 1:**
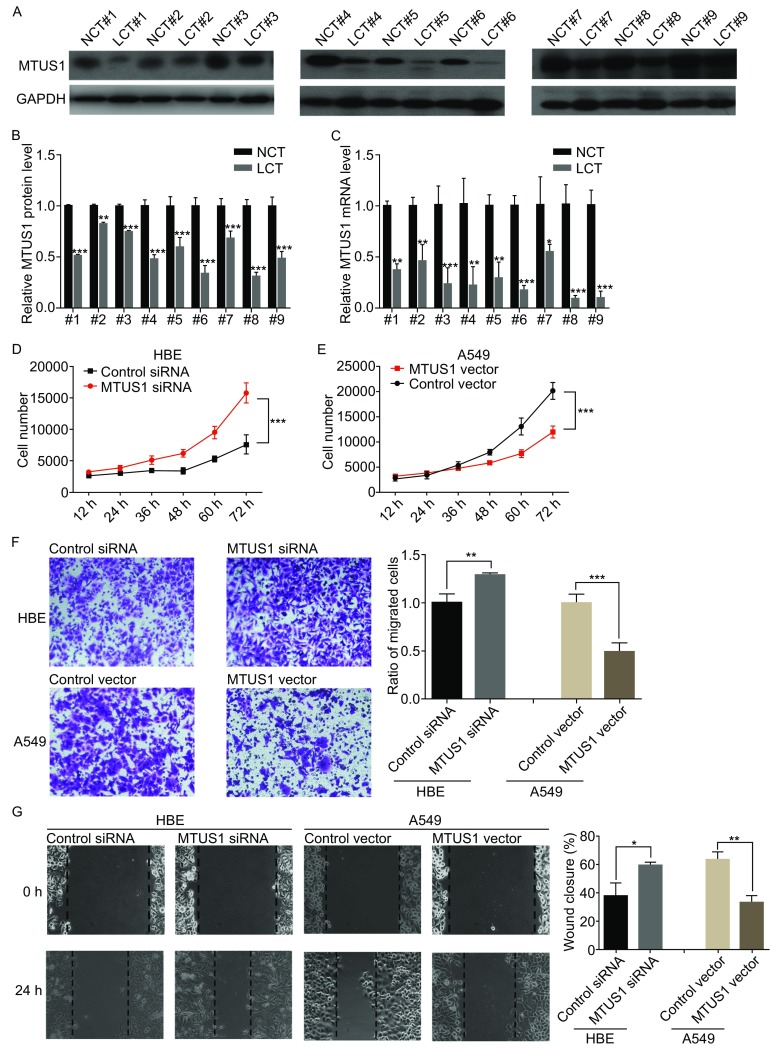
**MTUS1 functions as a tumor suppressor in lung cancer cells**. (A and B) Western blot analysis of MTUS1 protein levels in 9 pairs of lung cancer tissue (LCT) samples and adjacent noncancerous tissue (NCT) samples. (A) Representative image; (B) quantitative analysis. (C) Quantitative RT-PCR analysis of the relative expression levels of MTUS1 mRNA in 9 pairs of NCT and LCT samples. (D) Cell proliferation assay was performed 12, 24, 36, 48, 60, and 72 h after transfection of equal doses of MTUS1 siRNA/scrambled control siRNA into HBE cells. (E) Cell proliferation assay was performed 12, 24, 36, 48, 60, and 72 h after transfection of equal doses of MTUS1 vector/control vector into A549 cells. (F) Wound healing assays were performed 24 h after transfection of equal doses of MTUS1 siRNA/scrambled control siRNA into HBE cells or MTUS1 vector/control vector into A549 cells. Left panel: representative image; right panel: quantitative analysis of wound closure rates. (G) Transwell assays were performed 24 h after transfection of equal doses of MTUS1 siRNA or scrambled control siRNA into HBE cells or MTUS1 vector/control vector into A549 cells. Left panel: representative image; right panel: quantitative analysis. *, *P* < 0.05; **, *P* < 0.01; ***, *P* < 0.001

We first confirmed the expression of MTUS1 in human lung cancer tissues (The clinical features of the patients are listed in Table S1). After measuring the levels of MTUS1 protein expression in 9 pairs of lung cancer tissue samples and corresponding normal adjacent tissue samples, we found that MTUS1 protein levels were significantly lower in lung cancer tissues than in normal tissues (Fig. 1A and 1B). Correspondingly, MTUS1 mRNA levels were consistently downregulated in lung cancer tissues compared with normal tissues (Fig. 1C).

We next evaluated the biological functions of MTUS1 in lung cancer cell line A549. Normal human bronchial epithelial HBE was used as normal control. We designed 3 siRNA sequences targeting different human MTUS1 cDNA sites and transfected them into A549 cells (Fig. S1A). Then, we performed cell viability assay to determine the effects of MTUS1 on the proliferation of A549 and HBE. MTUS1 knockdown with siRNA significantly promoted HBE cell proliferation (Fig. 1D) and MTUS1 overexpression with MTUS1 vector inhibited A549 cell proliferation (Fig. 1E). We also used wound healing and transwell assays to investigate the effects of MTUS1 on the migration of A549 and HBE. HBE transfected with MTUS1 siRNA exhibited increased migration but A549 transfected with MTUS1 vector exhibited decreased migration (Fig. 1F and 1G). These results suggest that MTUS1 functions as a tumor suppressor and can suppress lung cancer cell proliferation and migration.

### Identification of conserved miR-19a/b target sites within the 3′-UTR of MTUS1

**Figure 2:**
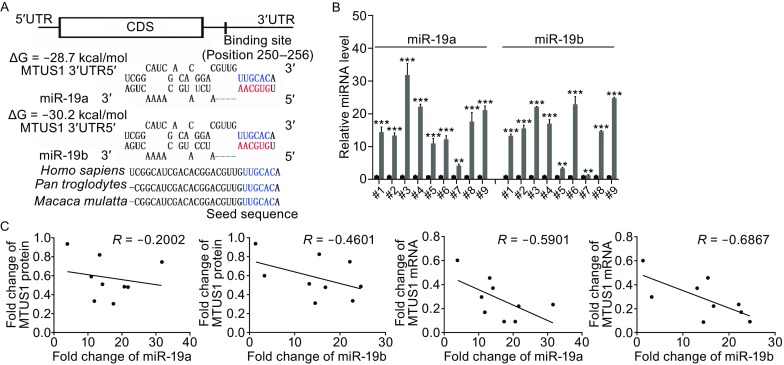
**Inverse correlation between miR-19a/b and MTUS1 levels in lung cancer tissue samples**. (A) Schematic description of the hypothetical duplexes formed by interactions between the MTUS1 3′-UTR (top) and miR-19a/b (bottom). The predicted free energy value of each hybrid is indicated. The seed recognition sites are denoted, and all nucleotides in these regions are highly conserved across several species. (B) Quantitative RT-PCR analysis of miR-19a/b levels in 9 pairs of NCT and LCT samples. (C) Pearson’s correlation scatter plot of the fold changes in miR-19a/b and MTUS1 protein/mRNA expression in lung cancer tissues. *, *P* < 0.05; **, *P* < 0.01; ***, *P* < 0.001

The mechanism underlying MTUS1 downregulation in lung cancer tissues remains largely unknown. One important mode of gene regulation is miRNA-mediated post-transcriptional mRNA transcript repression. Therefore, miRNAs likely play a biologically relevant role in regulating MTUS1 expression in lung cancer. TargetScan, miRanda, and PicTar were used in combination to identify potential miRNAs targeting MTUS1. MiR-19a/b were identified as candidate regulatory miRNAs of MTUS1 and were selected for further experimental verification. The predicted interactions between miR-19a/b and their target sites in the MTUS1 3′-UTR are illustrated in Fig. 2A. One overlapping hybrid between the MTUS1 3′-UTR and miR-19a or miR-19b was identified. The minimum free energy values of these hybridizations were −28.7 and −30.2 kcal/mol, which are well within the range of genuine miRNA-target pairs. Moreover, there was perfect base-pairing between the seed regions (The core sequences that encompass the first 2–8 bases of the mature miRNA) and cognate targets, and the miR-19a/b binding sequences in the MTUS1 3′-UTR were highly conserved across species.

### Detection of an inverse correlation between miR-19a/b and MTUS1 levels in lung cancer tissues

Because miRNAs generally exhibit expression patterns that contrast with those of their targets (Ambros, 2004; Bartel, 2004; He and Hannon, 2004), we investigated whether miR-19a/b expression levels were inversely correlated with MTUS1 expression levels in lung cancer. After determining the levels of miR-19a/b in 9 pairs of lung cancer tissues and corresponding normal adjacent tissues, we showed that miR-19a/b expression levels were consistently upregulated in lung cancer tissues (Fig. 2B). The inverse correlation between miR-19a/b levels and MTUS1 protein/mRNA levels was illustrated further using Pearson’s correlation scatter plots (Fig. 2C). Based on the results of the computational prediction and the detection of an inverse correlation between miR-19a/b expression and MTUS1 expression *in vivo*, it is very likely that miR-19a/b are involved in MTUS1 post-transcriptional regulation.

### Validation of MTUS1 as a direct target of miR-19a/b

**Figure 3:**
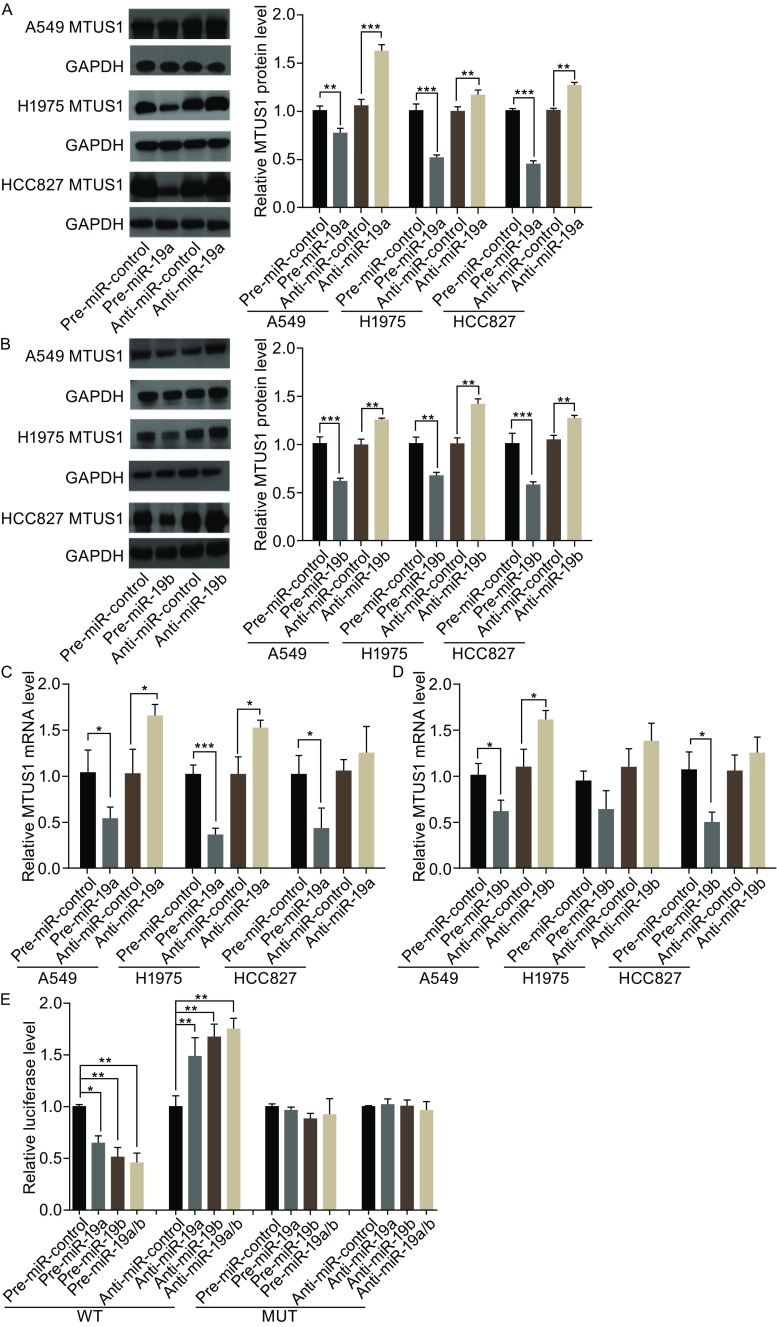
**Direct regulation of MTUS1 expression by miR-19a/b**. (A and B) Western blot analysis of MTUS1 protein levels in A549, H1975, and HCC827 cells transfected with pre-miR-control, pre-miR-19a/b, anti-miR-control or anti-miR-19a/b. (A) and (B) left: representative image; (A) and (B) right: quantitative analysis. (C and D) Quantitative RT-PCR analysis of MTUS1 mRNA levels in A549, H1975, and HCC827 cells transfected with pre-miR-control, pre-miR-19a/b, anti-miR-control or anti-miR-19a/b. (E) Direct recognition of the MTUS1 3′-UTR by miR-19a/b. 293T cells were co-transfected with firefly luciferase reporters containing either wild-type (WT) or mutant (Mut) miR-19a/b binding sites in the MTUS1 3′-UTR and pre-miR-control, pre-miR-19a/b, anti-miR-control, or anti-miR-19a/b. The cells were assayed using a luciferase assay kit 24 h after transfection. The results are displayed as the ratio of firefly luciferase activity in miR-19a/b-transfected cells to that in control cells. *, *P* < 0.05; **, *P* < 0.01; ***, *P* < 0.001

The correlation between miR-19a/b and MTUS1 was examined further by evaluating MTUS1 expression in three lung cancer cell lines (A549, H1975, and HCC827) in the setting of miR-19a/b overexpression or knockdown. In these experiments, miR-19a/b overexpression was achieved by transfecting cells with pre-miR-19a/b (synthetic RNA oligonucleotides mimicking miR-19a/b precursors), whereas miR-19a/b knockdown was achieved by transfecting cells with anti-miR-19a/b (chemically modified antisense oligonucleotides designed to specifically target mature miR-19a/b). As anticipated, cellular miR-19a/b levels were significantly increased in A549, H1975, and HCC827 cells when these cells were transfected with pre-miR-19a/b, whereas miR-19a/b levels were significantly decreased when these cells were transfected with anti-miR-19a/b (Fig. S2A and S2B). Consequently, MTUS1 protein expression was significantly inhibited by the introduction of miR-19a/b in A549, H1975 and HCC827 cells, while MTUS1 protein expression was significantly increased by the introduction of anti-miR-19a/b in these cells (Fig. 3A and 3B). To determine the level at which miR-19a/b influenced MTUS1 expression, we repeated the above experiments and examined MTUS1 mRNA expression after transfection. MTUS1 mRNA expression was decreased by miR-19a/b overexpression and increased by miR-19a/b knockdown in A549, H1975, and HCC827 cells (Fig. 3C and 3D). These results are consistent with the idea that animal miRNAs can decrease gene expression levels by regulating mRNA splicing or promoting mRNA degradation (Adams et al., 2014).

To determine whether the inhibitory effects exerted by miR-19a/b on MTUS1 expression are mediated via binding of miR-19a/b to their presumed target sites in the MTSU1 mRNA 3′-UTR, the entire MTSU1 3′-UTR containing the presumed miR-19a/b binding sites was fused to a reporter plasmid downstream of the firefly luciferase gene. The resulting plasmid was transfected into A549 cells along with a transfection control plasmid (β-gal) and pre-miR-19a/b, anti-miR-19a/b or scrambled negative control RNAs. As expected, miR-19a/b overexpression resulted in a ~40% reduction in luciferase reporter activity compared to cells treated with the pre-miR control, whereas miR-19a/b inhibition resulted in a 1.5-fold increase in reporter activity compared to cells transfected with the anti-miR control (Fig. 3E). Furthermore, we induced point mutations into the corresponding complementary sites in the MTUS1 3′-UTR to eliminate the predicted miR-19a/b binding sites. This mutated luciferase reporter was unaffected by miR-19a/b overexpression or knockdown (Fig. 3E). This finding suggests that the presumed binding sites strongly contribute to the abovementioned miRNA-mRNA interactions. In conclusion, these results suggest that miR-19a/b recognize and bind to the 3′-UTR of the MTUS1 mRNA transcript and inhibit MTUS1 translation.

As a single miRNA can target hundreds of genes, it is necessary to determine whether the effects of miR-19a/b on lung cancer cells are derived from miR-19a/b-mediated MTUS1 suppression. To investigate whether the regulation of cell proliferation, migration, and invasion by miR-19a/b is executed through a MTUS1-dependent manner, we co-transfected A549 cells with miR-19a/b mimic and the MTUS1-overexpression vector. Compared with cells transfected with miR-19a/b mimic and control vector, the cells transfected with both miR-19a/b mimic and MTUS1 vector exhibited a significantly lower proliferation rate (Fig. S3A), suggesting that miR-19a/b-resistant MTUS1 can attenuate the proliferative effect of miR-19a/b on lung cancer cells. Likewise, when A549 cells were simultaneously transfected with miR-19a/b mimic and the MTUS1 vector, MTUS1 dramatically attenuated the promotive effect of miR-19a/b on cell migration in transwell invasion assay and wound healing assay (Fig. S3B and S3C). Taken together, these results indicate that miR-19a/b may regulate the proliferation, migration, and invasion of lung cancer cells through a MTUS1-dependent manner.

### Co-treatment with miR-19a/b synergistically suppresses MTUS1 expression in lung cancer cells

**Figure 4:**
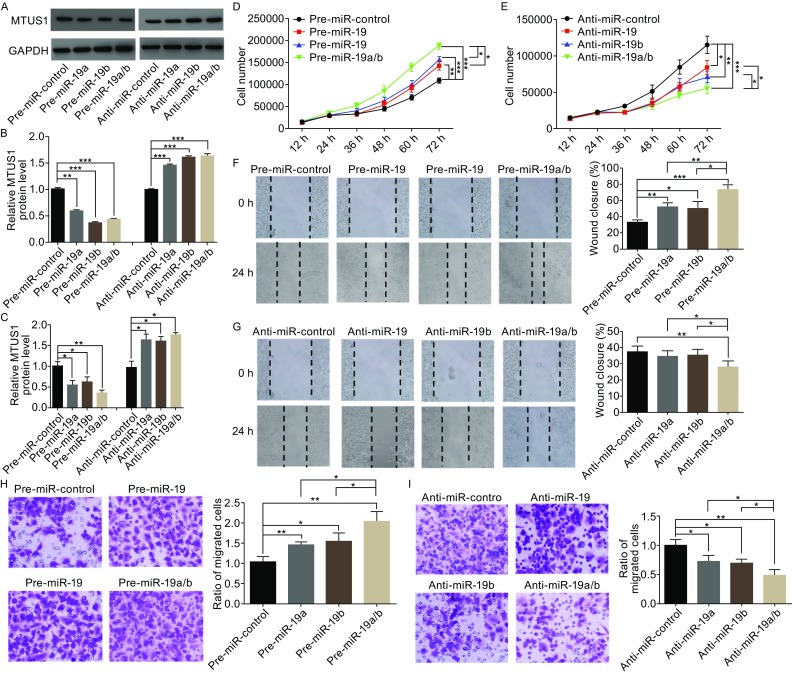
**Effects of miR-19a/b on lung cancer cell proliferation and migration**. (A and B) Western blot analysis of MTUS1 protein levels in A549 cells transfected with equal amounts of pre-miR-control (100 pmol), pre-miR-19a (100 pmol), pre-miR-19b (100 pmol) or pre-miR-19a/b (50 pmol each) or equal amounts of anti-miR-control (100 pmol), anti-miR-19a (100 pmol), anti-miR-19b (100 pmol) or anti-miR-19a/b (50 pmol each). (A) Representative image; B: quantitative analysis. (C) Quantitative RT-PCR analysis of MTUS1 mRNA levels in A549 cells transfected with equal amounts of pre-miR-control (100 pmol), pre-miR-19a (100 pmol), pre-miR-19b (100 pmol) or pre-miR-19a/b (50 pmol each) or equal amounts of anti-miR-control (100 pmol), anti-miR-19a (100 pmol), anti-miR-19b (100 pmol) or anti-miR-19a/b (50 pmol each). (D and E) CCK-8 viability assays were performed 12, 24, 36, 48, 60, and 72 h after transfection of equal amounts of pre-miR-control (100 pmol), pre-miR-19a (100 pmol), pre-miR-19b (100 pmol) or pre-miR-19a/b (50 pmol each) or equal amounts of anti-miR-control (100 pmol), anti-miR-19a (100 pmol), anti-miR-19b (100 pmol) or anti-miR-19a/b (50 pmol each) into A549 cells. (F and G) Wound healing assays were performed 24 h after transfection of equal amounts of pre-miR-control (100 pmol), pre-miR-19a (100 pmol), pre-miR-19b (100 pmol) or pre-miR-19a/b (50 pmol each) or equal amounts of anti-miR-control (100 pmol), anti-miR-19a (100 pmol), anti-miR-19b (100 pmol) or anti-miR-19a/b (50 pmol each) into A549 cells. Left panel: representative image; right panel: quantitative analysis of wound closure rates. (H and I) Transwell assays were performed 24 h after transfection of equal amounts of pre-miR-control (100 pmol), pre-miR-19a (100 pmol), pre-miR-19b (100 pmol) or pre-miR-19a/b (50 pmol each) or equal amounts of anti-miR-control (100 pmol), anti-miR-19a (100 pmol), anti-miR-19b (100 pmol) or anti-miR-19a/b (50 pmol each) into A549 cells. Left panel: representative image; right panel: quantitative analysis. *, *P* < 0.05; **, *P* < 0.01; ***, *P* < 0.001

MiR-19a/b belong to the same miRNA family and differ by only a single nucleotide at position 11. Because miR-19a/b display extensive sequence homology, they are thought to possess overlapping targets and have redundant functions. One goal of this study was to determine whether miR-19a/b function individually or synergistically. Equal amounts of pre-miR-19a (100 pmol), pre-miR-19b (100 pmol) or pre-miR-19a/b (50 pmol each) were transfected into A549 cells to overexpress miR-19a and/or miR-19b, respectively, and the reductions in MTUS1 levels were measured. Co-treatment with pre-miR-19a and pre-miR-19b successfully increased miR-19a/b levels (Fig. S2C and S2D) and enhanced MTUS1 protein and mRNA suppression compared to treatment with either pre-miR-19a or pre-miR-19b alone (Fig. 4A–C). These results indicated that the suppressive effects of miR-19a/b on MTUS1 protein and mRNA expression were not an individual effects but synergistic effects. Likewise, co-treatment with anti-miR-19a and anti-miR-19b successfully decreased miR-19a/b levels (Fig. S2C and S2D). While MTUS1 protein and mRNA expression was significantly increased in A549 cells transfected with anti-miR-19a (100 pmol) or anti-miR-19b (100 pmol), the greatest increase in expression occurred when anti-miR-19a and anti-miR-19b (50 pmol each) were transfected into A549 cells simultaneously (Fig. 4A–C). These results confirmed the synergistic effects of miR-19a/b on MTUS1 expression in lung cancer cells.

### The roles of miR-19a/b in regulating MTUS1 in lung cancer cells

To investigate the cellular phenotypes triggered by miR-19a/b-mediated MTUS1 downregulation, A549 cells were transfected with either pre-miR-19a/b or anti-miR-19a/b and then analyzed regarding changes in cell proliferation and migration. Cell viability assay showed that cell proliferation was significantly increased in A549 cells transfected with pre-miR-19a/b, which confirmed the hypothesis that miR-19a/b function as oncogenic miRNAs; in contrast, miR-19a/b knockdown had the opposite effect on A549 cell proliferation (Fig. 4D and 4E). Thus, the cell proliferation promoted by MTUS1 knockdown was similar to that elicited by miR-19a/b overexpression, indicating that miR-19a/b and MTUS1 exert contrasting effects on cell proliferation. Interestingly, although individual miR-19a or miR-19b overexpression/knockdown increases/decreases cell proliferation, simultaneous introduction/reduction of miR-19a and miR-19b exerted synergistic effects with respect to cell proliferation promotion/suppression compared to the effects exerted by miR-19a or miR-19b alone (Fig. 4D and 4E). These results suggest that miR-19a/b synergistically accelerate lung cancer cell proliferation. Furthermore, we evaluated the effects of miR-19a/b on lung cancer cell migration using wound healing and Transwell assays. During wound healing assay, more A549 cells migrated to the scratch in the cell monolayer when these cells were transfected with pre-miR-19a/b, while A549 cell mobility was significantly inhibited by anti-miR-19a/b transfection (Fig. 4F and 4G). In addition, Transwell assay revealed that the percentage of migrated cells was significantly higher among A549 cells transfected with pre-miR-19a/b and significantly lower among cells transfected with anti-miR-19a/b (Fig. 4H and 4I). Therefore, miR-19a/b and MTUS1 exerted opposing effects on cell migration. More importantly, both the wound healing and the Transwell assays indicated that simultaneous introduction of miR-19a and miR-19b exerted synergistic effects with respect to the promotion of cell migration compared to the effects exerted by miR-19a or miR-19b alone and that simultaneous inhibition of miR-19a and miR-19b decelerated cell migration more than inhibition of either miRNA alone (Fig. 4F–I). Taken together, our findings indicate that because miR-19a/b and MTUS1 exhibit contrasting expression patterns and exert contrasting biological effects in lung cancer cells, it is very possible that miR-19a/b synergistically modulate cell proliferation and migration in lung cancer cells by silencing MTUS1.

## DISCUSSION

MTUS1 was first identified as a tumor suppressor gene located on chromosome 8p21.3-22 in 2003, and it is also known as ATIP (AT2-receptor interacting protein), MTSG1 (mitochondrial tumor suppressor gene 1) GK1, and ATBP50 (Kinjo et al., 2000; Seibold et al., 2003; Wruck et al., 2005). The MTUS1 gene contains 17 coding exons distributed over 112 kb of genomic DNA. Alternative exon usage enables the generation of five transcripts (ATIP1, ATIP2, ATIP3a, ATIP3b, and ATIP4), each exhibiting different tissue distributions (Di Benedetto et al., 2006; Yu et al., 2009). All of these transcripts share a common 118 amino acid AT2-receptor interacting domain (ID), which suggests that all 5 MTUS1 transcripts interact with the AT2-receptor and induce atypical signal transduction pathways including at least three intracellular cascades involving protein phosphatase activation, nitric oxide regulation, and phospholipase A2 stimulation (Knowle et al., 2000). Additionally, recent studies have shown that MTUS1 loss or downregulation is associated with enhanced tumor proliferation, poor tumor differentiation, and poor prognosis in bladder cancer (Xiao et al., 2012), gastric cancer (Li et al., 2014a), salivary adenoid cystic carcinoma (Zhao et al., 2015), and other cancers (Louis et al., 2010; Louis et al., 2011; Ding et al., 2012; Varghayee et al., 2015). In addition, MTUS1 has been shown to interfere with activation of the ERK2 pathway and to inhibit proliferation of cells stimulated with growth factors, such as insulin, bFGF, PDGF, and EGF (Nouet et al., 2004; Zuern et al., 2010). Additionally, MTUS1 interference can result in inhibition of EB1 turnover at microtubule plus ends (Velot et al., 2015). Although several papers regarding the relationship between MTUS1 gene expression and cancer have been published, very little is known regarding the role and regulation of MTUS1 in lung cancer. In this study, we measured MTUS1 protein and mRNA levels in human lung cancer tissues and paired normal adjacent tissues and found that MTUS1 protein and mRNA levels were significantly decreased in cancer tissues, findings indicative of the potential tumor-suppressor functions of MTUS1 in lung cancer. Furthermore, MTUS1 knockdown by siRNA promoted HBE cell proliferation and migration. Similarly, MTUS1 overexpression by MTUS1 vector also suppressed A549 cell proliferation and migration. These indicate that MTUS1 negatively modulates lung cancer cell proliferation and migration to exert its tumor suppressor effects. However, the molecular mechanisms underlying MTUS1 regulation remain unclear and must be fully elucidated.

MiRNAs are important gene regulators and have been shown to be involved in the initiation, development, and metastasis of human cancers. During tumorigenesis, dysregulated miRNAs play either tumor-suppressor or oncogenic roles, depending on the functions of their target genes. Tumor-suppressor miRNAs usually repress oncogenes, and oncogenic miRNAs usually silence tumor suppressor genes. MiRNAs that are associated with human cancers are referred to as oncomirs. Two of the most famous oncomirs, miR-19a/b in the miR-17-92 miRNA cluster are involved in regulating of a wide variety of human cancers. In this study, we evaluated the expression patterns of miR-19a/b in lung cancer tissues and observed that the expression levels of both miR-19a and miR-19b were elevated in lung cancer tissues compared with those in paired normal adjacent tissues. We also showed that both miR-19a and miR-19b can function as promoters of lung cancer cell proliferation and migration. We found that miR-19a/b are co-upregulated in lung cancer and have concordant cellular functions, which allowed us to hypothesize that miR-19a/b may play important roles in lung carcinogenesis. Interestingly, MTUS1 was identified as a co-target of miR-19a/b by bioinformatics analysis, and an inverse correlation between miR-19a/b levels and MTUS1 levels was detected in lung cancer tissues, indicating that miR-19a/b overexpression may play a role in lung cancer progression by co-targeting MTUS1. Thus, this study delineated a novel regulatory network employing miR-19a/b and MTUS1 to regulate lung cancer cell fates. MTUS1 modulation by miR-19a/b may explain why miR-19a/b upregulation and MTUS1 downregulation during lung carcinogenesis promote cancer progression. In future studies, it will be important to determine how critical this newly identified pathway is for lung tumorigenesis.

Many miRNAs work in conjunction with one another to fine-tune gene expression on a global level. Thus far, most research regarding miRNAs has focused on the roles of individual miRNAs in the regulation of specific genes. It is important to study the cooperative roles played by multiple miRNAs, as this may provide us with new and more comprehensive insights regarding miRNA regulation within the cell. Studying the functions of miRNA family members seems to be a promising means of deciphering the cooperative effects of multiple miRNAs. MiRNA families comprise multiple miRNAs exhibiting primary sequence similarity to a specific seed sequence. Because this seed sequence plays an important role to mRNA target specificity, miRNA family members are thought to have extensive mRNA target overlap and redundant functions. One goal of this study was to determine whether miR-19a/b function individually or synergistically. In this study, miR-19a/b were shown to participate in cooperative MTUS1 repression. Thus, co-treatment with miR-19a and miR-19b synergistically promoted lung cancer cell growth and migration compared with treatment with miR-19a or miR-19b alone. These results indicate that individual miRNA family members displaying co-expression patterns can simultaneously and cooperatively repress a given target mRNA. However, why cells use such sophisticated and intricate mechanisms to regulate target gene expression is a fascinating question. Perhaps co-regulation serves as a fail-proof mode of miRNA regulation to ensure that when one member of a miRNA pair is disabled by mutations or dysfunction, the other is still available to perform its biological function. Cooperation between miRNA family members thus represents an interesting area of study that may change our perceptions regarding how miRNAs mediate gene regulation.

## CONCLUSIONS

In summary, this study not only elucidated the critical role of MTUS1 as a tumor suppressor in lung cancer but also explored the molecular mechanisms underlying MTUS1 regulation and identified miR-19a/b as direct upstream regulators of MTSU1 expression. This study has provided us with insights regarding the molecular mechanisms underlying lung carcinogenesis and has opened a new avenue for lung cancer treatment.

## MATERIALS AND METHODS

### Human tissue samples

Lung cancer tissue samples and paired normal adjacent tissue samples were obtained from patients undergoing surgical procedures at the Affiliated Drum Tower Hospital of Nanjing University Medical School (Nanjing, China). Tissue fragments were immediately frozen in liquid nitrogen at the time of surgery and stored at −80°C. The clinical features of the patients are listed in Table S1.

### Cell culture

The human lung adenocarcinoma epithelial cell lines A549, H1975, HCC827, and normal human bronchial epithelial cell line HBE were purchased from the Shanghai Institute of Cell Biology, Chinese Academy of Sciences (Shanghai, China). A549 cells were cultured in DMEM (GIBCO, Carlsbad, CA, USA) supplemented with 10% fetal bovine serum (FBS, GIBCO, Austria) in a humidified incubator at 37°C with 5% CO_2_. H1975, HCC827, and HBE cells were cultured in RPMI-1640 medium (GIBCO) supplemented with 10% FBS (GIBCO) in a humidified incubator at 37°C with 5% CO_2_.

### RNA isolation and quantitative RT-PCR

Total RNA was isolated from cultured cells or tissue samples using TRIzol Reagent (Invitrogen, USA). The total RNA concentration was determined using a BioPhotometer (Eppendorf, Germany). To quantify MTUS1 and GAPDH mRNA, 1 μg of total RNA was reverse-transcribed to cDNA using oligo(dT) 18 primers (TaKaRa, Dalian, China) and avian myeloblastosis virus (AMV) reverse transcriptase (TaKaRa, Dalian, China). The reaction conditions were as follows: 42°C for 60 min and 70°C for 10 min. Real-time PCR was then performed using the RT product, SYBR Green dye (Invitrogen, USA) and specific primers for MTUS1 and GAPDH (Table S2). The reaction mixture was incubated at 95°C for 5 min, followed by 40 cycles of 95°C for 30 s, 60°C for 30 s, and 72°C for 1 min. After the reactions, C_T_ values were determined by setting a fixed threshold. The ratio of MTUS1 mRNA to GAPDH mRNA, which served as an internal control, was calculated using the equation 2^−△△CT^, in which △△C_T_ = (C_T MTUS1_ − C_T GAPDH_)_tumor_ − (C_T MTUS1_ − C_T GAPDH_)_control_.

MiRNA quantification assay was performed using TaqMan miRNA probes (Applied Biosystems, Foster City, CA, USA), according to the manufacturer’s instructions. Briefly, 1 μg of total RNA was reverse-transcribed to cDNA using AMV reverse transcriptase and a stem-loop RT primer (Applied Biosystems). The reaction conditions were as follows: 16°C for 30 min, 42°C for 30 min, and 85°C for 5 min. Real-time PCR was conducted using a TaqMan PCR kit and an Applied Biosystems 7500 Sequence Detection System (Applied Biosystems). The reaction was incubated in a 96-well optical plate at 95°C for 10 min, followed by 40 cycles of 95°C for 15 s and 60°C for 1 min. All reactions were run in triplicate. After the reaction, cycle threshold (C_T_) data were calculated using fixed threshold settings, and the mean C_T_ was determined from the triplicate PCR results. The comparative C_T_ method was used to determine relative miRNA levels. The ratio of miR-19a/b to U6, which served an internal control, was calculated using the equation 2^−△△CT^, in which △△C_T_ = (C_T miR-19a/b_ − C_T U6_)_tumor_ − (C_T miR-19a/b_ − C_T U6_)_control_.

### Plasmid construction and siRNA interference assay

Mammalian expression plasmids designed to specifically express the full-length open reading frame (ORF) of the human MTUS1 gene were purchased from Genescript (Nanjing, China). An empty plasmid served as a negative control (control vector). Three siRNAs targeting different sites of the human MTUS1 were designed and synthesized by Invitrogen (Carlsbad, CA, USA). A scrambled siRNA (Invitrogen) was included as a negative control. siRNAs were transfected into A549 cells using Lipofectamine 2000 (Invitrogen,Carlsbad, Calif), according to the manufacturer’s instructions. Total RNA or protein was isolated 48 h after transfection. MTUS1 mRNA and protein expression levels were assessed by quantitative RT-PCR and Western blotting, respectively.

### MiRNA overexpression and knockdown

MiRNA overexpression was achieved by transfecting cells with a miRNA mimic, whereas knockdown was achieved by transfecting cells with a miRNA inhibitor. Synthetic RNA molecules, including pre-miR-19a and pre-miR-19b (miRNA mimics), anti-miR-19a and anti-miR-19b (miRNA inhibitors), and scrambled negative control RNAs (pre-miR-control and anti-miR-control), were purchased from GenePharma (Shanghai, China). A549, H1975, and HCC827 cells were seeded in 6-well plates and transfected with Lipofectamine 2000 on the following day, when the cells were approximately 80% confluent. For miRNA overexpression, equal amounts of pre-miR-19a (100 pmol), pre-miR-19b (100 pmol) or pre-miR-19a/b (50 pmol each) were used. For miRNA knockdown, equal amounts of anti-miR-19a (100 pmol), anti-miR-19b (100 pmol) or anti-miR-19a/b (50 pmol each) were used. After 6 h, the medium was changed to DMEM or RPMI-1640 supplemented with 2% FBS. The cells were harvested 48 h after transfection for total RNA or protein isolation, respectively.

### Luciferase reporter assay

The entire 3′-untranslated region (3′-UTR) of the human MTUS1 was amplified by PCR using human genomic DNA as a template. The PCR products were inserted into the p-MIR-reporter plasmid (Ambion, Austin, TX, USA). The insertion was confirmed to be correct by DNA sequencing. To test binding specificity, the sequences that interact with the seed sequence of miR-19a/b were mutated (from TTGCAC to AACGTG), and the mutant MTUS1 3′-UTR was inserted into an equivalent luciferase reporter plasmid. For the luciferase reporter assays, 293T cells were seeded in 24-well plates and co-transfected with 0.5 μg of firefly luciferase reporter plasmid, 0.5 μg of β-galactosidase (β-gal) expression plasmid (Ambion,Austin, Tex), and equal amounts (25 pmol) of miR-19a/b mimics, inhibitors, or scrambled negative control RNAs using Lipofectamine 2000 (Invitrogen). The β-gal plasmid was used as a transfection control. Cells were harvested 24 h after transfection and analyzed for luciferase activity using a luciferase assay kit (Promega, Madison, WI, USA).

### Protein isolation and Western blotting

Cells or tissues were lysed in a RIPA lysis buffer (50 mmol/L Tris-HCl, 150 mmol/L NaCl, 0.1% SDS, 1% NP-40, 0.25% sodium deoxycholate, and 1 mmol/L EDTA, pH 8.0). Proteins were separated by SDS-PAGE. After electrophoresis, the proteins were electrotransferred to PVDF membranes and then blocked with 5% skim milk for 1 h. The membranes were then incubated with a primary antibody against MTUS1 (Abcam, Cambridge, Mass) and GAPDH (Santa Cruz Biotechnology, Santa Cruz, CA) at 4°C for 12 h. After three washes in TBST, the membranes were incubated with horseradish peroxidase-conjugated secondary antibody for 1 h at room temperature. After three washes, the membranes were incubated with the SuperSignal West Pico chemiluminescence substrate (Pierce Biotechnology, USA).

### Cell viability assay

A549 cells were plated at a density of 2 × 10^4^ cells per well in 96-well plates and then incubated overnight in DMEM supplemented with 10% FBS. Cells were collected at 12, 24, 48, and 72 h post-transfection. After transfection, 10 mL of Cell CountingKit-8 solution (#C0038, Beyotime, Jiangsu, China) was added to the appropriate test wells and incubated for 1 h. Absorbance was measured at a wavelength of 450 nm.

### Transwell invasion assay

The migration ability of A549 cells was tested in a Transwell Boyden Chamber (6.5-mm, Costar, USA). The polycarbonate membranes (8-µm pore size) on the bottom of the upper compartment of the Transwell chamber were coated with 1% human fibronectin (R&D systems 1918-FN, USA). The cells were harvested 24 h after transfection and suspended in FBS-free DMEM. Then, the cells were added to the upper chamber (4 × 10^4^ cells/well). At the same time, 0.5 mL of DMEM with 10% FBS was added to the lower compartment, and the Transwell-containing plates were incubated for 12 h in a 5% CO_2_ atmosphere saturated with H_2_O. After incubation, the cells that had entered the lower surface of the filter membrane were fixed with 4% paraformaldehyde for 25 min at room temperature, washed 3 times with distilled water and stained with 0.1% crystal violet in 0.1 mol/L borate and 2% ethanol for 15 min at room temperature. Cells remaining on the upper surface of the filter membrane (non-migrant) were scraped off gently with a cotton swab. Images of the lower surfaces (with migrant cells) were captured by a photomicroscope (5 fields per chamber) (BX51 Olympus, Japan), and the cells were counted blindly.

### Wound healing assays

Cell migration was assessed in a classical wound healing assay, with some minor modifications. Briefly, cells were seeded in 6-well plates and transfected when they were attached. After transfection, the cells were allowed to grow to confluence. Then, the cell layer was gently wounded using a plastic pipette tip (P200) and rinsed with PBS before the culture medium was replaced. The bottoms of the wells were marked to indicate where the initial images of the wounded area were captured. After 24 h of incubation, images (10×) of the same areas were recorded using a photo microscope (BX51 Olympus, Japan), and wound closure was processed using Image-Pro Plus 6.0.

### Statistical analysis

All Western blotting, wound healing assay, and Transwell assay images are representative of at least three independent experiments. Quantitative RT-PCR and luciferase reporter assays were performed in triplicate, and each experiment was repeated at least three times. The results are presented as the mean ± SD. Differences between groups were calculated using Student’s *t*-test, and *P* < 0.05 was considered statistically significant.

## Electronic supplementary material

Below is the link to the electronic supplementary material.

Supplementary material 1 (PDF 678 kb)
