# Supplementary material for: Oncogenic miR-19a and miR-19b co-regulate tumor suppressor MTUS1 to promote cell proliferation and migration in lung cancer
Source: Protein Cell. 2017 Mar 31;8(6):455–66. doi: 10.1007/s13238-017-0393-7 (PMC5445029; doi:10.1007/s13238-017-0393-7)
Supplement: Supplementary file 1 — Supplementary material 1 (PDF 678 kb) [file 13238_2017_393_MOESM1_ESM.pdf]

miR-19a and miR-19b co-regulate MTUS1 to promote cell proliferation and migration in lung cancer

Yuanyuan Gu[1,*], Shuoxin Liu[2,*], Xiaodan Zhang[1], Guimin Chen[2], Hongwei Liang[1], Mengchao Yu[1], Zhicong Liao[3], Yong Zhou[3], Chen-Yu Zhang[1], Tao Wang[3,#], Chen wang[1,#] , Junfeng Zhang[1,#], Xi Chen[1,#]

Additional file1: Table 1

	Age	Gender	Tumor subtype	Pathological Stage
Case #1	67	Female	Adenocarcinoma	III A
Case #2	70	60	Adenocarcinoma	I B
Case #3	72	Male	Squamous cell carcinoma	
Case #4	70	Male	Adenocarcinoma	I
Case #5	65	Female	Adenocarcinoma	III A
Case #6	59	Female	Adenocarcinoma	III A
Case #7	60	Male	Squamous cell carcinoma	I B
Case #8	59	Male	Adenocarcinoma	I
Case #9	76	Female	Adenocarcinoma	

Additional file 2: primer set

MTUS1	forward	5’-GGACTAGTCCTCCCCAAAGTCCACAG-3’
	reverse	5’-CCCAAGCTTATTTCATTCACACCCCCC-3’
GAPDH	forward	5’-GATATTGTTGCCATCAATGAC-3’
	reverse	5’-TTGATTTTGGAGGGATCTCG-3’

Additional file 3: Figure S1. Evaluation of MTSU1 knockdown efficiency in A549 cells.

A

siRNA name	Sequence
MTUS1 siRNA1	5'-GCCUCCCUUUCAGAAAUUATT-3'
MTUS1 siRNA2	5'-GCCCAAAGAUGCUGCUUUATT-3'
MTUS1 siRNA3	5'-GGGCUCAUGUUCACUUGAUTT-3'

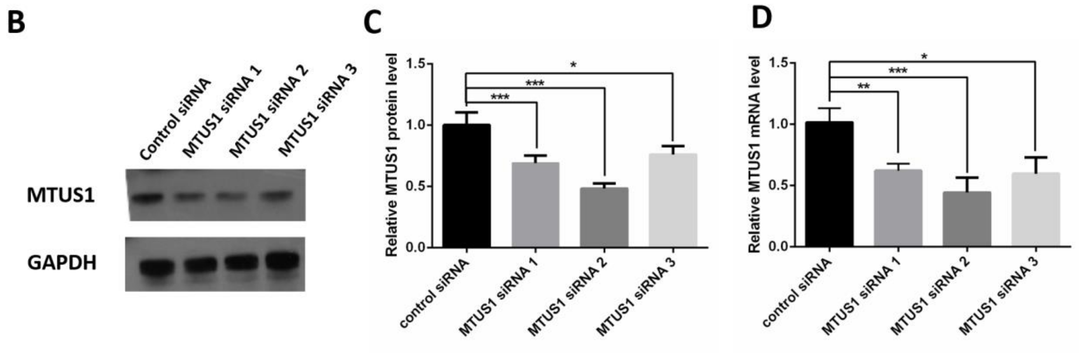

(A) Sequences of three siRNAs designed for targeting MTUS1. **(B and C)** Western blotting analysis of MTUS1 protein levels in A549 cells transfected with three different siRNAs or scrambled control siRNA. B: representative image; C: quantitative analysis. **(D)** Quantitative RT-PCR analysis of MTUS1 mRNA levels in A549 cells transfected with three different MTUS1 siRNAs or scrambled control siRNA. *, $P < 0.05$; **, $P < 0.01$; ***; $P < 0.001$.

Additional file 4: Figure S2. Evaluation of miR-19a/b overexpression and knockdown efficiency in A549 cells.

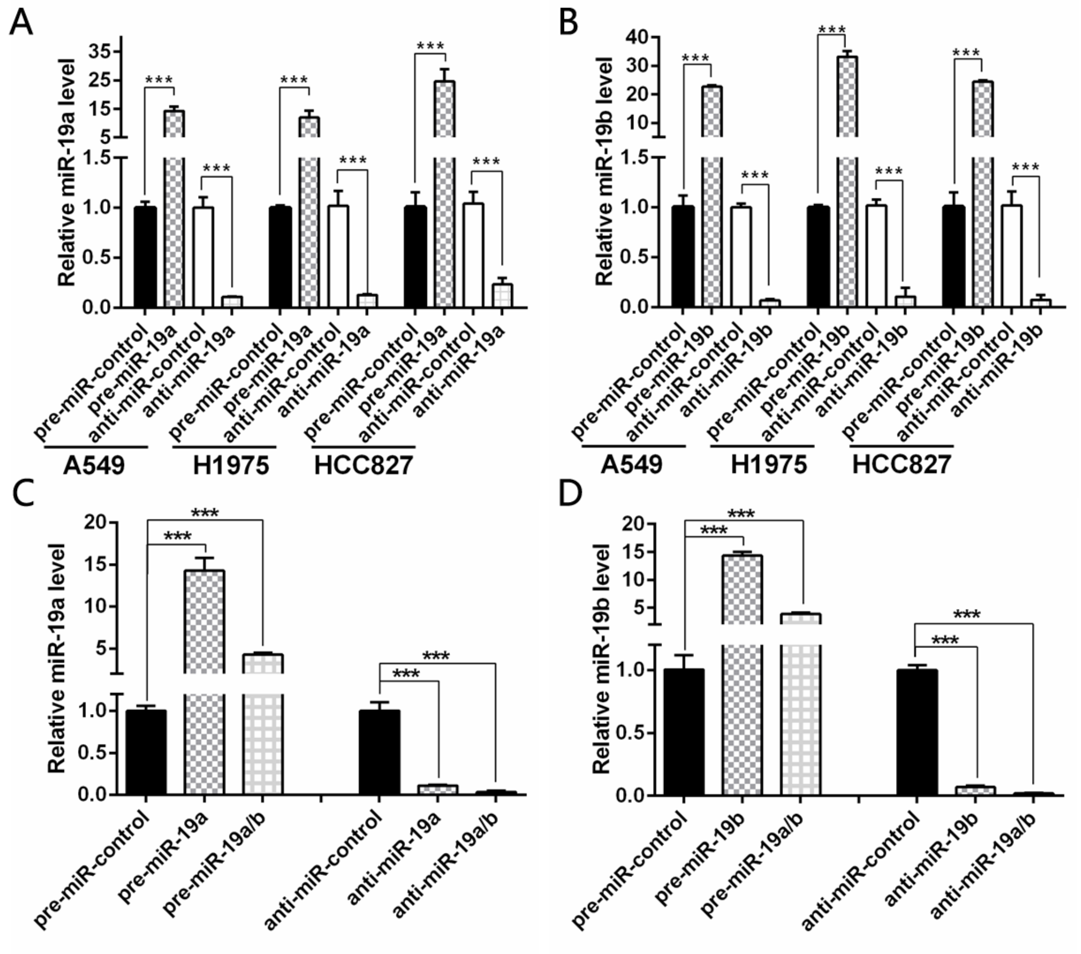

(A and B) Quantitative RT-PCR analysis of miR-19a/b levels in A549, H1975 and HCC827 cells transfected with pre-miR-control, pre-miR-19a/b, anti-miR-control or anti-miR-19a/b. **(C and D)** Quantitative RT-PCR analysis of miR-19a/b levels in A549 cells transfected with equal amounts of pre-miR-control (100 pmol), pre-miR-19a (100 pmol), pre-miR-19b (100 pmol) or pre-miR-19a/b (50 pmol each) or equal amounts of anti-miR-control (100 pmol), anti-miR-19a (100 pmol), anti-miR-19b (100 pmol) or anti-miR-19a/b (50 pmol each). *, $P < 0.05$; **, $P < 0.01$; ***, $P < 0.001$.

Additional file 5: Figure S3. Co-effect of miR-19a/b and MTUS1 on the proliferation and migration of lung cancer cells.

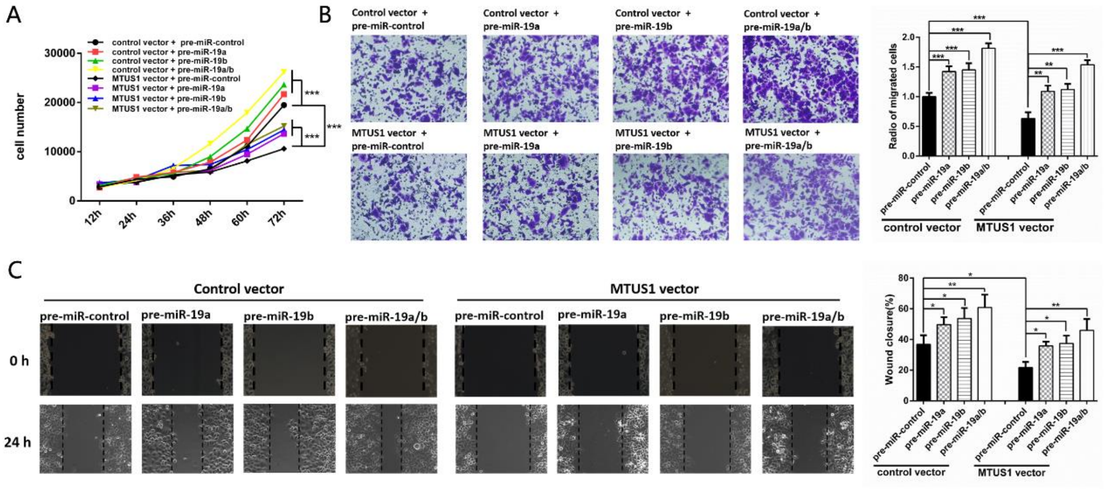

(A) CCK-8 viability assays were performed 12, 24, 36, 48, 60 and 72 h after transfection of pre-miR-control, pre-miR-19a, pre-miR-19b, pre-miR-19a/b plus control vector or MTUS1 vector into A549 cells. **(B)** Transwell assays were performed 24 h after transfection of pre-miR-control, pre-miR-19a, pre-miR-19b, pre-miR-19a/b plus control vector or MTUS1 vector into A549 cells. Left panel: representative image; Right panel: quantitative analysis. **(C)** Wound healing assays were performed 24 h after transfection of pre-miR-control, pre-miR-19a, pre-miR-19b, pre-miR-19a/b plus control vector or MTUS1 vector into A549 cells. Left panel: representative image; Right panel: quantitative analysis. *, $P < 0.05$; **, $P < 0.01$; ***, $P < 0.001$.
